# Clinical Characteristics and Predictors of Glycemic Control During the First 24 Months After Diagnosis of Type 1 Diabetes

**DOI:** 10.3390/biomedicines14030690

**Published:** 2026-03-17

**Authors:** Selina Löffler, Fabio Frigo, Daniel Hochfellner, Elke Fröhlich-Reiterer, Faisal Aziz, Hanna Kubesch, Thomas Pieber, Harald Sourij, Felix Aberer

**Affiliations:** 1 Division of Endocrinology and Diabetology, Department of Internal Medicine, Medical University of Graz, 8036 Graz, Austria; selina.loeffler@stud.medunigraz.at (S.L.); daniel.hochfellner@medunigraz.at (D.H.); faisal.aziz@medunigraz.at (F.A.); thomas.pieber@medunigraz.at (T.P.); ha.sourij@medunigraz.at (H.S.); 2 Department of Endocrinology, Rheumatology and Acute Geriatrics, Klinik Ottakring, 1160 Vienna, Austria; fabio.frigo@gesundheitsverbund.at; 3 Department of Pediatrics and Adolescent, Medicine Division of General Pediatrics, Medical University of Graz, 8036 Graz, Austria; elke.froehlich-reiterer@medunigraz.at; 4 Cardiometabolic Trials Unit, Medical University of Graz, 8036 Graz, Austria; 5 Department of Food Chemistry and Toxicology, University of Vienna, 1090 Vienna, Austria; hanna.kubesch@yahoo.de

**Keywords:** type 1 diabetes, glycemic control (HbA_1c_), autoantibodies, risk predictors, C-peptide, treatment setting, retrospective cohort study

## Abstract

**Background**: Long-term glycemic control in type 1 diabetes (T1D) varies substantially among affected individuals, but the role of baseline characteristics at diagnosis and their association with later glycemic control remain incompletely understood. Identifying early predictors of glycemic control may facilitate timely, individualized therapeutic interventions. **Methods**: We retrospectively analyzed electronic health records of individuals with newly diagnosed T1D between 2001 and 2022 to assess anthropometric and metabolic parameters at the first presentation of the condition across age groups and determine predictors of glycated hemoglobin (HbA_1c_) trajectories over 24 months. The multicentric cohort, which comprised people who were diagnosed with T1D in the Austrian federal state of Styria, was classified as children (<10 years), adolescents (10–18 years) or adults (≥18 years). Variables of interest included demographic and anthropometric data, positivity and titers of diabetes-specific autoantibodies, treatment setting (inpatient/outpatient), and presence and severity of diabetic ketoacidosis (DKA). **Results**: The cohort consisted of 281 individuals (23.1% were children, 41.3% were adolescents, and 35.6% were adults at T1D diagnosis; 46.6% were female). In the unadjusted analyses, younger age (age < 18 years), female sex, and receiving treatment in a general ward were associated with higher HbA_1c_ levels over 24 months. However, after adjustment for important covariates, only younger age remained a significant predictor of inferior glycemic control over 24 months, emphasizing the importance of structured, age-appropriate follow-up care. **Conclusions**: Younger age at T1D diagnosis independently predicts suboptimal glycemic trajectories over the first two years after T1D onset. Early identification may enable targeted, age-specific interventions to improve long-term outcomes.

## 1. Introduction

Type 1 diabetes (T1D) is a T cell–mediated autoimmune disease characterized by a dysregulated immune response leading to the destruction of pancreatic β-cells [1]. Globally, T1D is among the most common autoimmune metabolic disorders in children and adolescents, affecting 1 in 250 individuals by the age of 17 years [2]. In Germany, the number of newly diagnosed cases increases by approximately 3200 per year [2]. In Austria, there is an alarming increase in the prevalence of diabetic ketoacidosis (DKA) at T1D onset, especially in children < 2 years [3]. According to projections, the global number of individuals living with T1D is estimated to increase from approximately 8.4 million in 2021 to between 13.5 and 17.4 million by 2040, representing a relative increase of 60% to 107% over this period [4]. The etiology of T1D is believed to involve a combination of genetic predisposition, virus-induced immune responses, and environmental factors [1]. At the time of diagnosis, a significant proportion of β-cells are already destroyed, resulting in diabetic ketoacidosis (DKA) in 25–40% of cases, often requiring intensive care treatment [5,6,7]. Over one-third of affected individuals are diagnosed at an advanced stage, already presenting with severe metabolic disturbances [7]. This highlights the importance of early recognition of T1D and of predictors for future suboptimal glycemic outcomes [8].

Several predictors of insufficient glycemic control are already described in the literature, including DKA at diagnosis, overweight and obesity, comorbidities, first-born status, baseline HbA_1c_ > 9.5%, a higher titer of GAD-A antibodies, low socioeconomic status, family history of diabetes, non-White ethnicity, and poor adherence to treatment or diet [9,10,11].

Suboptimal HbA_1c_ levels, often accompanied by metabolic, social and mental impairments, reduce quality of life and increase morbidity and mortality [12,13]. The knowledge of predictors impacting later glycemic control after T1D diagnosis is important in regard to identifying those with insufficient blood glucose control or complications at an early stage. This enables intensified monitoring and therapeutic strategies from the earliest stages of the disease. If certain factors that influence subsequent blood glucose control are known, treatment can be individually adapted. For example, more individualized insulin therapy or closer monitoring may be appropriate for certain groups of people living with diabetes. Furthermore, affected people might be encouraged for motivation for consistent treatment and beneficial lifestyle changes when predictors of prospective glucose control are individually defined. Understanding predictors might also promote the development of new treatment approaches and improve clinical decision-making.

The aim of this study is, therefore, to investigate predictive factors for suboptimal metabolic control within the first 2 years in people with newly diagnosed T1D in order to identify early indicators of a potentially adverse disease course. Additional objectives include characterizing this population and identifying associations or differences in laboratory values, medical interventions and other parameters across subgroups during diabetes onset and during the further course of the disease.

## 2. Materials and Methods

This retrospective analysis encompassed data of people with T1D at any age being in care in an outpatient clinic of a secondary and tertiary care center. The study population was identified by a diagnosis-based health record search performed by the Institute for Medical Informatics (Medical University of Graz). People with the diagnosis of T1D within their medical records who visited the outpatient clinic between 1 January 2001 and 31 December 2022 were included and followed up according to HbA_1c_ for 2 further years.

Initially, 1606 individuals were identified. After manual review and exclusion of cases with incomplete data, a final sample of 281 people with sufficiently available follow-up data was included in the analysis.

The time of initial diagnosis was determined through a manual review of electronic records, covering entries from 2001 through 31 December 2022 in order to ensure a 2-year follow-up period. People younger than 18 years at diagnosis who were treated at the department of pediatrics were also included, as were those whose diagnosis or treatment occurred at other facilities within the Styrian Hospital Association (KAGes) indicating a national multicenter setting.

Demographic and medical data were retrospectively extracted from the electronic medical record system. The index visits were physical presentations at medical institutions of the specific hospital association (KAGes), either at outpatient clinics, emergency rooms or intensive care units due to the very first manifestation of hyperglycemia in response to T1D onset. Collected parameters at the index visit were categorized as follows: demographic data (age, sex, body weight, height, BMI), disease-specific data (ketoacidosis yes/no [defined as pH < 7.3], setting of primary care [outpatient, general ward or intensive care unit], pediatric or adult setting), laboratory values (pH, HbA_1c_, C-peptide, glucose, C-peptide/glucose ratio [CGR], creatinine, glomerular filtration rate [GFR], C-reactive protein [CRP], total cholesterol, low-density lipoprotein cholesterol [LDL-C], triglycerides, proinsulin) as well as diabetes-specific antibodies (islet cell antibodies [IC-A], insulin antibodies [IA-A], tyrosine phosphatase antibodies [IA-2-A], glutamate decarboxylase antibodies [GAD-A] and zinc-transporter antibodies [ZnT8-A]). HbA_1c_ values as well as C-peptide and glucose were retrospectively extracted from the medical records at the time points of 3, 6, 12 and 24 months after the index visit.

### 2.1. Ethical Considerations

All data were pseudonymized using people’s IDs only for privacy protection. This study was approved by the Ethics Committee of the Medical University of Graz (EK-No. 36-208 ex 23/24).

### 2.2. Statistical Analysis

Statistical analyses were performed using IBM SPSS Statistics, version 30. Continuous variables are presented as the mean ± standard deviation, while categorical variables are presented as frequencies and percentages. Group comparisons were conducted using independent samples t-tests, chi-squared tests and one-way ANOVA, as appropriate. Pearson’s correlation coefficients were used to assess the relationships between the baseline variables. To evaluate the course of glycemic control, HbA_1c_ levels at 3, 6, 12 and 24 months were analyzed using linear mixed-effects models with repeated measures and random intercepts. Baseline HbA_1c_ at diagnosis was included as a covariate. In the linear mixed-effects models, estimates represent the mean difference in HbA_1c_ between comparison groups or per unit increase in a continuous predictor. Positive estimates indicate higher HbA_1c_ values compared to the respective reference category.

For each predefined clinical and immunological factor (age group, sex, presence of diabetic ketoacidosis, initial treatment setting, C-peptide–glucose ratio tertiles, and number of positive diabetes-specific autoantibodies), separate models were fitted and adjusted for age, sex, and baseline HbA_1c_. Body mass index (BMI) was not included in the adjusted models due to a high proportion of missing data.

For each factor, both the global association with HbA_1c_ (*p*-value) and the interaction between time and the factor (p for interaction, p_int_) were assessed to evaluate whether the effect on HbA_1c_ changed over time. Type III tests with Satterthwaite’s approximation were applied for significance testing, and Bonferroni correction was used for post hoc comparisons. A *p*-value < 0.05 was considered statistically significant.

Generative AI (ChatGPT, based on the GPT-5.3 model) was used exclusively for language editing and not for content generation or data analysis.

## 3. Results

A total of 281 people were included in the analysis. Based on age at diagnosis, 65 individuals (23.1%) were classified as children (<10 years), 116 (41.3%) were classified as adolescents (10–18 years), and 100 (35.6%) were classified as adults (≥18 years). The mean age of the total cohort was 18.0 ± 13.0 years (range 1–79), and 46.6% of the included people were female. The mean body mass index (BMI) was 18.6 ± 4.9 kg/m^2^. Baseline characteristics of the specific age-based populations are indicated in Table 1.

### 3.1. Baseline Comparisons

#### 3.1.1. Age Groups

All three age groups differed significantly in weight (*p* < 0.001), height (*p* < 0.001) and, subsequently, BMI (*p* < 0.001) as well as creatinine levels (*p* < 0.001). Furthermore, adults exhibited significantly lower glucose levels at disease onset compared to children and adolescents (children vs. adults: *p* < 0.001; adolescents vs. adults: *p* = 0.049). HbA_1c_ and presence of DKA were comparable in all age groups.

#### 3.1.2. HbA_1c_

In the total population, HbA_1c_ at diagnosis was significantly positively correlated with serum creatinine (*p* = 0.014), total cholesterol (*p* < 0.001) and triglycerides (*p* < 0.001). Negative correlations were observed between HbA_1c_ and BMI (*p* = 0.009), pH (*p* < 0.001) and the C-peptide/glucose ratio (CGR *p* < 0.001).

#### 3.1.3. C-Peptide and C-Peptide to Glucose Ratio (CGR)

The CGR showed a moderate positive correlation with pH (*p* = 0.004). Negative correlations were found with HbA_1c_ (*p* < 0.001) and creatinine (*p* = 0.011).

#### 3.1.4. Sex Aspect

Regarding sex-specific differences, male individuals had higher body weight (*p* = 0.020), height (*p* < 0.001), creatinine (*p* = 0.011) and eGFR (*p* = 0.047), whereas women had higher C-peptide levels (*p* = 0.042) and lower pH values (*p* = 0.035) at T1D diagnosis. No statistically significant differences were found between sexes in terms of type of care at diagnosis, presence of DKA, HbA_1c_ or the number of positive autoantibodies.

#### 3.1.5. Diabetic Ketoacidosis (DKA)

A total of 82 cases of DKA at diabetes onset were observed in this cohort study, with 18 in children, 39 in adolescents and 25 in adults, respectively. The presence of DKA was associated with lower body weight (*p* = 0.010) and height (*p* = 0.026) and several metabolic parameters at baseline, including higher HbA_1c_, CRP, creatinine, LDL-C, total cholesterol, and triglycerides and lower C-peptide, CGR, proinsulin, and eGFR (all *p* < 0.05). DKA was primarily treated in the intensive care setting (97.3%). Additionally, a significant association was found between DKA and the number of positive autoantibodies (*p* = 0.027), though no linear trend was observed (*p* = 0.445).

#### 3.1.6. Type of Care (Outpatient, General Ward, Intensive Care Unit)

Individuals treated in the intensive care unit (ICU) presented with higher levels of HbA_1c_, creatinine, glucose, and triglycerides and lower levels of C-peptide, CGR, pH, BMI and proinsulin compared to those managed in the outpatient setting (all *p* < 0.05). Management type also varied significantly with age (*p* < 0.001), with younger people more likely to receive treatment in the general ward or intensive care and older individuals more frequently managed on an outpatient basis. A linear trend supported this association (*p* < 0.001). Moreover, the type of care was significantly associated with the presence of DKA (*p* < 0.001).

#### 3.1.7. Autoantibodies

**Figure 1:**
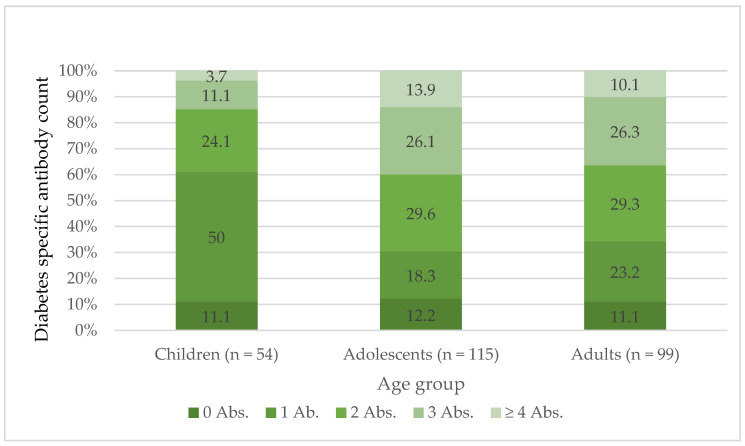
Total diabetes-specific autoantibody distribution (0–5 antibodies (Abs.) by age group (children, adolescents and adults) in percent [%] at baseline.

The presence of four or more positive autoantibodies was associated with higher body weight, height, and creatinine and lower levels of C-peptide and triglycerides compared to people with no positive autoantibodies at diagnosis (all *p* < 0.05). A significant association was observed between age at diagnosis and the number of positive diabetes-specific autoantibodies (*p* = 0.007). Children most frequently had 0 or 1 positive autoantibody (11.1% and 50%, respectively), while ≥4 autoantibodies were rare (3.7%), as seen in Figure 1. In contrast, adolescents and adults more often presented with multiple autoantibodies, with ≥4 positive markers found in 13.8% and 10.1%, respectively. A test for linear trend confirmed that higher age at onset was associated with a greater number of positive autoantibodies (*p* = 0.025).

### 3.2. Predictors of HbA_1c_ Progression over 24 Months with Linear Mixed Model

**Figure 2:**
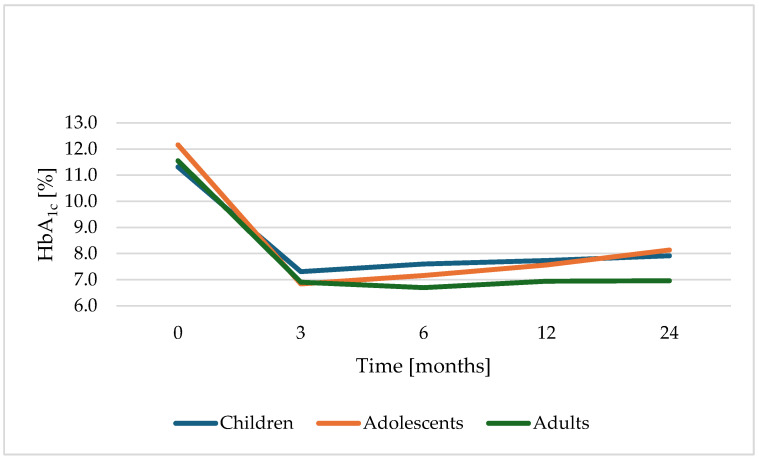
Mean HbA_1c_ trajectory during the first 24 months after T1D diagnosis by age group, including baseline HbA_1c_.

Across all age groups, mean HbA_1c_ decreased significantly in the first six months and stabilized thereafter [HbA_1c_ at index visit: 11.75% [CI: 11.46–12.04], at 3 months: 6.98% [CI: 6.84–7.11], at 6 months 7.12% [CI: 6.97–7.27], at 12 months 7.42% [CI: 7.27–7.56], and at 24 months 7.75% [CI: 7.59–7.91] (*p* < 0.001)]. Figure 2 shows a descriptive visualization of the HbA_1c_ trajectory over 24 months in three specific age groups.

#### 3.2.1. Unadjusted Analysis of Baseline Variables on HbA_1c_

Management upon initial diagnosis

After adjustment for only baseline HbA_1c_, during follow-up, outpatient management was associated with significantly lower HbA_1c_ values compared with people admitted to the general ward (*p* = 0.012); however, there was no significant difference between general ward care and intensive care regarding HbA_1c_ trajectories (*p* = 1.000). On average, individuals admitted to the general ward at diagnosis showed an HbA_1c_ difference of +0.54% compared to outpatient care (*p* = 0.009) during the 2 years of follow-up, also shown in Table 2. For people in ICU, this effect was slightly smaller at +0.51% in comparison to outpatient therapy (*p* = 0.078).

Age and sex

**Figure 3:**
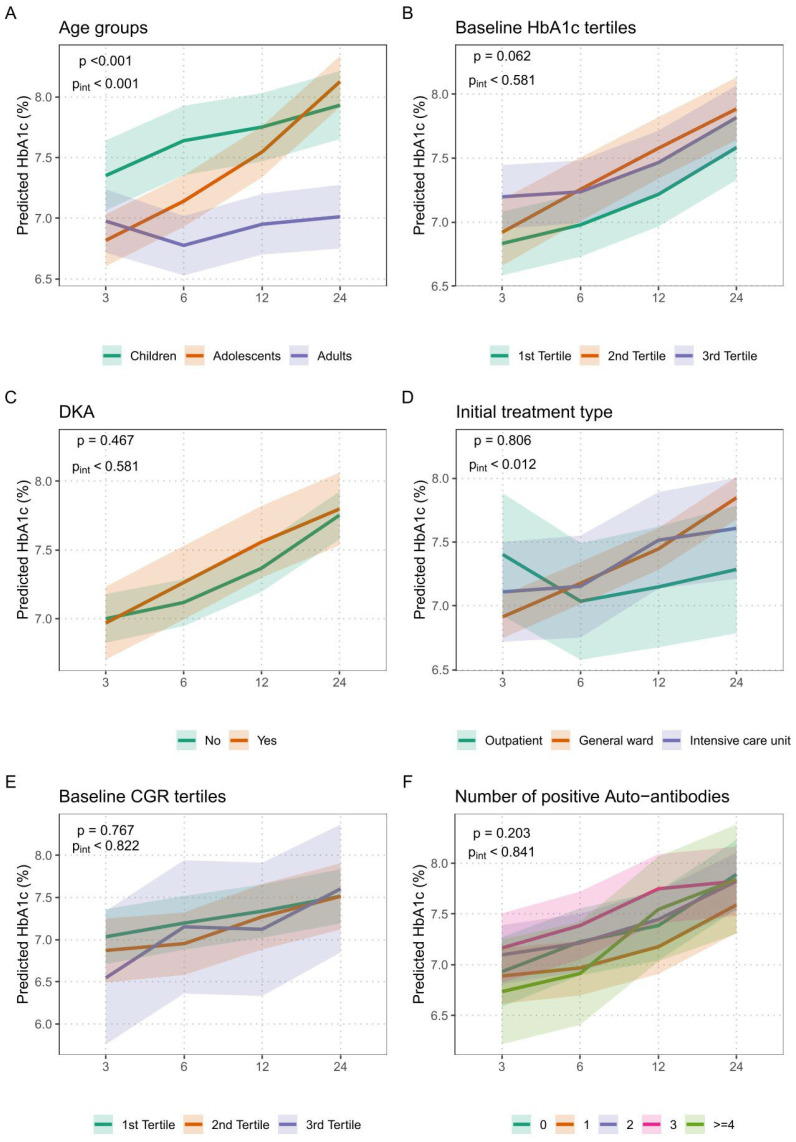
Adjusted predictors of glycemic control (HbA_1c_ trajectory) in interaction with time estimated using linear mixed-effects models over the 24-month follow-up period. All models are adjusted for age, sex, and baseline HbA_1c_. (**A**): Association of age group at diagnosis (Children, Adolescents, Adults) with glycemic trajectory. (**B**): Association of baseline HbA_1c_ tertiles with HbA_1c_ course over 24 months (Tertile 1: 5.45–10.66%, Tertile 2: 10.70–12.76%, Tertile 3: 12.80–18.80%). (**C**): Association of diabetic ketoacidosis (DKA; Yes vs. No) at baseline with HbA_1c_ progression. (**D**): Effect of initial treatment setting (Outpatient, General Ward, Intensive Care Unit) on HbA_1c_ course. (**E**): Association of C-peptide to glucose ratio (CGR) tertiles at diagnosis with HbA_1c_ over time (Tertile 1: ≤0.47, Tertile 2: 0.48–1.22, Tertile 3: 1.23–5.00). (**F**): Association of the number of positive diabetes-specific autoantibodies (0 to ≥4) with HbA_1c_ trajectory.

Age at first manifestation and our predefined age groups (children, adolescents and adults) showed a significant association with HbA_1c_ levels over the first 24 months post-T1D diagnosis. Adolescents demonstrated the most unfavorable glycemic trajectory, with the steepest increase in HbA_1c_, whereas adults maintained the lowest and most stable levels throughout follow-up, as shown in Figure 2 and Figure 3A. Throughout the entire study period, average HbA_1c_ levels of female individuals were 0.22% higher than males at all measurement times (*p* = 0.034).

Antibody count and titer

The number of positive diabetes-specific autoantibodies (GAD-A, IA-2-A, ZnT8-A, IA-A) at diagnosis showed no significant association with average HbA_1c_ levels over the 24-month follow-up period (*p* = 0.399).

The titer of glutamic acid decarboxylase (GAD-A) antibodies had no significant association with HbA_1c_ progression. People with higher GAD-A Ab levels did not tend to develop suboptimal HbA_1c_ values (*p* = 0.312) across all age groups. Also, the quantitative value of the other antibodies (ZnT8-A, IA-2-A, IA-A) were not significantly associated with HbA_1c_ trajectories.

C-Peptide to Glucose ratio

A lower number of positive diabetes-specific autoantibodies (GAD-A, IA-2-A, ZnT8-A, IA-A) at diagnosis was significantly associated with higher average C-peptide to glucose ratios over the 24-month follow-up period (*p* = 0.045), indicating better preserved β-cell function.

Tertiles (tertile 1: CGR ≤ 0.47, tertile 2: 0.48–1.22, tertile 3: 1.23–5.00) for CGR were created to evaluate whether higher CGR values were associated with more favorable HbA_1c_ trajectories. However, this analysis revealed no significant effect of CGR group on HbA_1c_ levels across follow-up (*p* = 0.573). Taken together, these findings indicate that baseline CGR tertile did not significantly predict the further course of HbA_1c_ over 24 months.

Diabetic ketoacidosis

In the total cohort, neither the presence of any diabetic ketoacidosis (defined as pH < 7.3) showed a significant association with HbA_1c_ levels over 24 months after initial manifestation (*p* = 0.331) nor the manifestation with a severe DKA (defined as pH < 7.0) (*p* = 0.273). All the unadjusted parameters are summarized in Table 2.

#### 3.2.2. Additional Unadjusted Outcomes

Tertiles for baseline HbA_1c_ % (1: 5.45–10.66%; 2: 10.70–12.76%; 3: 12.80–18.80%) were created to assess the association with HbA_1c_ trajectories over time. However, this analysis revealed no significant effect of different baseline HbA_1c_ tertiles on subsequent HbA_1c_ levels (*p* = 0.542), indicating that baseline HbA_1c_ does not influence later glycemic control.

A CRP > 5 mg/L at the time of T1D diagnosis was not significantly associated with a higher HbA_1c_ in the linear mixed-effects model over 24 months (*p* = 0.086).

The presence of impaired renal function at baseline (defined as eGFR < 60 mL/min/1.73 m^2^ in individuals aged ≥ 18 years) was not significantly associated with suboptimal glycemic control over 24 months. Although a trend towards higher HbA_1c_ levels was observed in adult individuals with reduced eGFR (*p* = 0.088), this did not reach statistical significance.

The baseline LDL-C level was not significantly associated with overall HbA_1c_ levels during the 24-month follow-up period (*p* = 0.531). Also, the baseline total cholesterol level was not significantly associated with higher overall HbA1c levels during the 24-month follow-up period (*p* = 0.660). The baseline triglyceride level was not significantly associated with overall HbA_1c_ levels during the 24-month follow-up period (*p* = 0.390).

#### 3.2.3. Adjusted Analysis of Predictors on HbA_1c_ over 24 Months

In the adjusted linear mixed-effects models, each predefined clinical and immunological variable was analyzed separately, with adjustment for age, sex, and baseline HbA_1c_. Two *p*-values were reported: the global *p*-value (*p*), assessing the overall association of the factor with HbA_1c_, and the interaction *p*-value (pint), evaluating whether the effect of the factor on HbA_1c_ changed over time.

Among all investigated factors, age group showed a highly significant global effect (*p* < 0.001) and a significant interaction with time (pint < 0.001), as shown in Figure 3A. This indicates that age at diagnosis not only affected mean HbA_1c_ levels but also influenced their trajectory over the 24-month follow-up period. Children and adolescents exhibited higher HbA_1c_ values across all time points, with adolescents showing the steepest deterioration.

Initial treatment setting was also associated with a significant interaction effect (pint = 0.012), though the global effect was not statistically significant (*p* = 0.806). This suggests that, while the overall HbA_1c_ means did not differ significantly by treatment setting, the HbA_1c_ trajectories over time did: individuals initially managed in outpatient care maintained more stable glycemic control compared to those admitted to general wards or intensive care.

In contrast, baseline HbA_1c_ tertiles showed no significant global association (*p* = 0.062) and no interaction with time (pint = 0.581), indicating that baseline HbA_1c_ levels were not predictive of glycemic outcomes over 24 months.

Similarly, the presence of diabetic ketoacidosis (DKA) at diagnosis was not significantly associated with HbA_1c_ course, with both the global effect (*p* = 0.467) and interaction (pint = 0.581) remaining non-significant, as shown in Figure 3C.

The C-peptide to glucose ratio (CGR) at diagnosis did not significantly predict the course of HbA_1c_ either, as evidenced by a *p* = 0.767 and pint = 0.822, also shown in Figure 3E.

Finally, the number of positive diabetes-specific autoantibodies was not significantly associated with HbA_1c_ outcomes (*p* = 0.203 and pint = 0.841), suggesting limited prognostic utility of immunological status in the early disease course, as seen in Figure 3F.

## 4. Discussion

This retrospective multicenter cohort study aimed to identify early predictors of glycemic trajectories during the first 24 months after diagnosis of type 1 diabetes (T1D). The key and clinically most relevant finding is that younger age at diagnosis—particularly onset before 18 years—was the only independent predictor of suboptimal glycemic control in fully adjusted models. All other clinical, metabolic, and immunological factors lost independent significance after adjustment, underscoring age at onset as the dominant determinant of early glycemic outcomes.

Children and adolescents showed consistently higher HbA_1c_ values over the entire follow-up period compared with adults, with adolescents displaying the most unfavorable trajectories. This finding is in strong agreement with large registry and cohort studies demonstrating deterioration of glycemic control during adolescence and young adulthood [13].

First, early-onset T1D is frequently associated with a more aggressive autoimmune process and more rapid β-cell destruction. Younger children tend to have lower residual C-peptide levels, reflecting diminished endogenous insulin secretion and reduced capacity to buffer glycemic excursions. In the Diabetes Control and Complications Trial Research Group (1993), preservation of C-peptide was strongly associated with improved glycemic outcomes and reduced complications [14]. Greenbaum et al. (2012) demonstrated that younger age at diagnosis is linked to lower stimulated C-peptide levels [15]. Similarly, Barker et al. (2014) reported an age-dependent decline in β-cell function in new-onset T1D [16]. Registry analyses from the Type 1 Diabetes Exchange further show that children diagnosed at younger ages have higher mean HbA_1c_ values compared with young adults [17].

Second, developmental physiology complicates glycemic management. Young children exhibit unpredictable food intake, variable activity, and heightened insulin sensitivity, increasing hypoglycemia risk. Fear of hypoglycemia among caregivers has been associated with higher HbA_1c_ [18]. During puberty, increased growth hormone and sex steroids induce insulin resistance, contributing to deterioration in glycemic control [19].

Third, psychosocial factors are critical. Glycemic outcomes in childhood are strongly influenced by family functioning and parental involvement. Greater family conflict is associated with higher HbA_1c_ [20]. Parental stress and depressive symptoms correlate with poorer adherence and metabolic control. Survey analyses indicated that psychiatric stress among patients and their caregivers is directly associated with a younger age at diabetes diagnosis, supporting our observation that earlier onset may be linked to poorer glycemic control [21,22].

Female sex at diagnosis was associated with higher HbA_1c_ levels in unadjusted analyses. Sex-related differences in glycemic control have been reported previously, with women often exhibiting higher HbA_1c_ and complication risk [23]. However, in the present study, sex did not remain an independent predictor after adjustment, suggesting that its effect is largely mediated by age-related and anthropometric factors.

Despite clear associations between diabetic ketoacidosis (DKA) at diagnosis and adverse metabolic parameters at baseline, neither the presence nor the severity of DKA independently predicted HbA_1c_ trajectories. This contrasts with earlier reports linking DKA at onset to worse long-term outcomes [24]. Our findings suggest that, within a healthcare system providing structured acute management and standardized follow-up, the negative prognostic impact of DKA may be largely reversible once metabolic stability is restored.

Neither the number nor titers of diabetes-specific autoantibodies (IC-A, GAD-A, IA-2-A, ZnT8-A, IA-A) were associated with glycemic control over 24 months. While a higher autoantibody burden correlated with lower C-peptide–glucose ratios—indicating reduced residual β-cell function—this did not translate into worse HbA_1c_ outcomes during the first 24 months after diagnosis. Previous studies have reported conflicting results regarding the prognostic role of autoantibodies, with some suggesting more aggressive disease in individuals with multiple antibodies [25,26], whereas others indicate slower progression in GAD-A-positive individuals [27]. Our data support the interpretation that autoantibody profiles are essential for diagnosis and disease classification but have limited value for predicting early glycemic trajectories.

Inflammatory markers, lipid parameters, renal function, and baseline HbA_1c_ tertiles were not independently associated with HbA_1c_ trajectories. Although these variables are relevant for long-term cardiovascular and renal risk, they appear to play a minor role in shaping early glycemic outcomes after T1D onset.

The dominance of age at diagnosis as a predictor of early glycemic control has direct clinical implications. Children and adolescents with newly diagnosed T1D should be considered a high-risk group for early suboptimal glycemic control, independent of disease severity at presentation or immunological markers. Intensified, age-adapted diabetes education, psychosocial support, and close follow-up during the first years after diagnosis are essential to mitigate long-term risks associated with sustained hyperglycemia.

This study is limited by its retrospective design, which is subject to missing data, misclassification, and residual confounding, and does not allow causal inference. The selection of patients in our study was based on the T1D diagnosis as generated by the registry search, which served as the primary source for cohort identification. In clinical practice in Austria, in cases where islet autoantibodies are not detectable, the diagnosis of type 1 diabetes is typically established based on absent or markedly reduced C-peptide levels in combination with the clinical presentation and after exclusion of other forms of diabetes, such as monogenic diabetes.

In addition, we would like to emphasize the temporal context of our cohort. Our analysis includes individuals diagnosed with diabetes starting in 2001, a time when autoantibody panels were more limited than today because fewer islet autoantibodies were known and routinely tested. As a consequence, some individuals classified as antibody-negative at that time might have been positive if broader antibody panels (e.g., including ZnT8) had been available. This may partly explain the slightly higher proportion of individuals without detectable autoantibodies in our cohort.

Another limitation that has to be addressed is that the cohort originates from a single Austrian hospital network with a predominantly White population and universal healthcare access, potentially limiting generalizability. Data on socioeconomic status, diabetes education intensity, insulin delivery modalities, and glucose monitoring technologies were not consistently available. Furthermore, the 24-month follow-up may be insufficient to detect longer-term effects of immunological and metabolic factors on glycemic control. Nevertheless, this study provides robust real-world evidence identifying age at diagnosis as the central determinant of early HbA_1c_ trajectories in T1D.

## 5. Conclusions

This retrospective multicenter cohort study found that age at diagnosis was the strongest and only factor showing both a significant overall association and a significant time interaction with glycemic control during the first 24 months after T1D onset. Individuals diagnosed before the age of 18 years had consistently higher HbA_1c_ levels throughout the follow-up period, indicating a substantially increased risk of suboptimal long-term glycemic control.

No statistically significant overall associations with HbA_1c_ trajectories were observed for sex, baseline HbA_1c_ tertile, initial treatment setting (outpatient, general ward, or intensive care unit), presence or severity of diabetic ketoacidosis at diagnosis, C-peptide–glucose ratio tertiles, or the number of positive islet autoantibodies. However, the initial treatment setting showed a significant interaction with time, indicating differences in HbA_1c_ trajectories over follow-up despite comparable overall HbA_1c_ levels.

Overall, these findings emphasize the dominant role of younger age at diagnosis as an independent predictor of suboptimal glycemic control in people with newly diagnosed T1D. This highlights the need for age-specific diabetes education, intensified follow-up, and early therapeutic support, particularly for children and adolescents.

## Figures and Tables

**Table 1:** Baseline characteristics of the specific age-based populations in total numbers (n) and percent (%); BMI, body mass index; CGR, C-peptide–glucose ratio; CRP, C-reactive protein; DKA, diabetic ketoacidosis; HbA_1c_, glycated hemoglobin; LDL-C, low-density lipoprotein cholesterol.

	Children (n = 65)	Adolescents (n = 116)	Adults (n = 100)	Total Population (n = 281)
Age in years (n)	7 ± 2 (65)	13 ± 2 (116)	32 ± 13 (100)	18.5 ± 13.3 (281)
BMI in kg/m^2^ (n)	15.07 ± 2.67 (51)	18.15 ± 4.40 (98)	22.89 ± 4.33 (53)	18.62 ± 4.91 (202)
HbA_1c_ in % (n)	11.32 ± 4.23 (65)	12.16 ± 4.63 (116)	11.55 ± 4.82 (99)	11.75 ± 4.63 (280)
C-peptide in ng/mL (n)	1.18 ± 1.42 (26)	1.30 ± 1.51 (52)	0.81 ± 0.97 (95)	1.16 ± 1.23 (179)
Glucose in mg/dL (n)	464.46 ± 176.5 (65)	411.64 ± 190.64 (116)	353.48 ± 168.78 (99)	403.34 ± 184.16 (280)
C-peptide–glucose ratio (n)	1.11 ± 0.93 (13)	1.35 ± 1.47 (55)	1.16 ± 1.03 (51)	1.24 ± 1.24 (119)
pH value (n)	7.34 ± 0.10 (57)	7.31 ± 0.14 (107)	7.32 ± 0.15 (75)	7.32 ± 0.14 (239)
CRP in mg/L (n)	3.63 ± 7.69 (65)	4.42 ± 9.98 (114)	7.03 ± 15.4 (83)	5.21 ± 11.54 (262)
Creatinine in mg/dL (n)	0.67 ± 0.16 (64)	0.85 ± 0.25 (116)	1.03 ± 0.33 (98)	0.87 ± 0.29 (278)
Total cholesterol in mg/dL (n)	177.08 ± 42.27 (65)	190.66 ± 50.3 (111)	196.53 ± 96 (72)	188.8 ± 65.53 (248)
LDL-C in mg/dL (n)	87.2 ± 42.76 (65)	90.58 ± 34.61 (45)	97.5 ± 35.28 (56)	94.08 ± 35.18 (160)
Triglycerides in mg/dL (n)	285.37 ± 311.65 (65)	257.18 ± 203.48 (110)	234.08 ± 381.39 (73)	257.77 ± 293.61 (248)
Proinsulin in pmol/L (n)	9.9 (1)	15.6 ± 8.91 (2)	12.02 ± 12.14 (65)	12.09 ± 11.93 (68)
Sex—female n (%)	37 (56.9)	50 (43.1)	44 (44)	131 (46.6)
DKA at diagnosis n (%)	18 (27.7)	39 (33.6)	25 (25)	82 (29.2)
Management outpatient n (%)	0 (0)	2 (1.7)	31 (31)	33 (11.7)
Management general ward n (%)	58 (89.2)	97 (83.6)	56 (56)	211 (75.1)
Management intensive care unit n (%)	7 (10.8)	17 (14.7)	13 (13)	37 (13.2)
Positive autoantibodies 0 n (%)	6 (11.1)	14 (12.2)	11 (11.1)	31 (11)
Positive autoantibodies 1 n (%)	27 (50)	21 (18.3)	23 (23.2)	71 (25.3)
Positive autoantibodies 2 n (%)	13 (24.1)	34 (29.6)	29 (29.3)	76 (27)
Positive autoantibodies 3 n (%)	6 (11.1)	30 (26.1)	26 (26.3)	62 (22.1)
Positive autoantibodies ≥ 4 n (%)	2 (3.7)	16 (13.9)	10 (10.1)	28 (10)

**Table 2:** Unadjusted predictors of glycemic control (HbA_1c_ trajectory) over 24 months derived from linear mixed-effects models. Estimates represent the mean difference in HbA_1c_ compared with the respective reference category or per unit increase in the predictor. Positive estimates indicate higher HbA_1c_ values, whereas negative estimates indicate lower HbA_1c_. BMI, body mass index; CGR, C-peptide to glucose ratio; CI, confidence interval; CRP, C-reactive protein; DKA, diabetic ketoacidosis; GAD-A, glutamic acid decarboxylase autoantibodies; eGFR, estimated glomerular filtration rate; IA-2-A, tyrosine phosphatase autoantibodies; IA-A, insulin autoantibodies; IC-A, islet cell antibodies; ICU, intensive care unit; LDL-C, low-density lipoprotein cholesterol; SE, standard error; ZnT8-A, zinc-transporter 8 antibodies.

Unadjusted Predictors of HbA_1c_ Trajectory over 24 Months:	Estimate	SE:	95%-CI:	*p*-Value:
Ketoacidosis (pH < 7.3) yes/no	2.956	3.018	[−2.96; 8.76]	*p* = 0.331
Severe ketoacidosis with pH < 7	1.766	1.410	[−4.53; 1.00]	*p* = 0.273
ICU vs. general ward	−0.028	0.161	[−5.716; 5.104]	*p* = 1.000
ICU vs. outpatient	−0.510	2.495	[−11.587; 0.429]	*p* = 0.078
General ward vs. outpatient	−0.539	0.180	[−0.972; –0.105]	*p* = 0.009
Total autoantibodies without IC-A (0–4)	-	-	-	*p* = 0.399
Total autoantibodies with IC-A (0–5)	-	-	-	*p* = 0.734
Titer IA-A Abs. (U/mL)	+0.00005	0.00018	[−0.00037; 0.00046]	*p* = 0.896
Titer ZnT8-A Abs. (U/mL)	−0.237	0.426	[−1.084; 0.610]	*p* = 0.580
Titer IA-2-A Abs. (U/mL)	−0.000274	0.000183	[−0.000640; 0.000091]	*p* = 0.180
Titer GAD-A Abs. (U/L)	−0.00027	0.00027	[−0.00082; 0.00027]	*p* = 0.312
Titer ICA Abs. (0–4)	-	-	-	*p* = 0.923
Age	−0.034	0.0086	[−0.051; −0.017]	*p* < 0.001
Age Groups	-	-	-	*p* < 0.001

## Data Availability

The datasets generated and/or analyzed during the current study are not publicly available due to data protection and patient confidentiality regulations but are available from the corresponding author on reasonable request and with permission of the relevant ethics committee.

## Abbreviations

The following abbreviations are used in this manuscript:

Abs.	antibodies
ANOVA	analysis of variance
BMI	body mass index
CGR	C-peptide to glucose ratio
CI	confidence interval
CRP	C-reactive protein
DKA	diabetic ketoacidosis
eGFR	estimated glomerular filtration rate
GAD-A	glutamate acid decarboxylase autoantibodies
GFR	glomerular filtration rate
HbA_1c_	glycated hemoglobin
IA-2-A	tyrosine phosphatase autoantibodies
IA-A	insulin autoantibodies
IC-A	islet cell antibodies
ICU	intensive care unit
KAGes	Steiermärkische Krankenanstaltengesellschaft
LDL-C	low-density lipoprotein
SE	standard error
SPSS	Statistical Package for the Social Sciences
T1D	type 1 diabetes mellitus
ZnT8-A	zinc-transporter isoform 8 autoantibodies
